# Differences in Well-Being at School Between Young Students With and Without a Refugee Background

**DOI:** 10.1007/s10578-024-01690-6

**Published:** 2024-04-05

**Authors:** Hanneke Leeuwestein, Elisa Kupers, Marieke Boelhouwer, Marijn van Dijk

**Affiliations:** 1 Department of Developmental Psychology, University of Groningen, Groningen, Netherlands; 2 Department of Inclusive and Special Needs Education, University of Groningen, Groningen, Netherlands; 3 Molendrift, Center for Youth Mental Healthcare, Groningen, Netherlands

**Keywords:** Refugees, Well-being, Mental health, Trauma, Post-traumatic stress, Primary education

## Abstract

Students with a refugee background are a vulnerable group in education. Adverse experiences and unsafe circumstances that they encounter prior, during and after their flight can place a great burden on their mental health and psychological well-being. Little is known about the psychological well-being of young refugee students in kindergarten and early years of primary school. The current study examined the psychological well-being of 4- to 8-year-old students with a refugee background residing in the Netherlands (*n* = 136), compared to Dutch peers without a refugee background (*n* = 406). Primary school teachers completed three questionnaires which assessed multiple indicators of their students’ psychological well-being: Strengths and Difficulties Questionnaire (SDQ), Social-Emotional Questionnaire (SEV) and Risk and Protective factors Trauma Observation School Situations (RaPTOSS). In line with the hypothesis, results showed overall lower psychological well-being among refugee students compared to non-refugee students. Teachers observed more total difficulties in socio-emotional functioning, anxious and mood disturbing behavior, ADHD symptoms, problematic social behavior and post-traumatic stress symptoms (small effects), and less developed trauma protective factors and prosocial behavior (medium effects) among students with a refugee background compared to their non-refugee peers. However, the findings also demonstrated that half of the refugee students did not have any scores that fall in the clinical range of the psychological and behavioral problems assessed. The results underline the need to promote protective factors such as positive self-image, self-regulation skills, safety and relations in the classroom and prosocial behavior among students with a refugee background.

The number of forcibly displaced people worldwide is now higher than ever before with 110 million people on the move to seek safety [1]. Approximately 40% of them are children, resulting in many schools nowadays having students with a refugee background in their classrooms [1]. While settling into a new school environment in a new country can be a struggle, it also creates opportunities for improved psychological well-being and integration in the host country [2–4]. However, teachers with young refugee students in their classroom express their worries about these students’ well-being, with underlying trauma or distress often being suspected as reason for worrisome behavior and learning difficulties [5].

Most refugees witnessed or experienced dramatic life changing events prior, during and after their flight journey [6, 7]. These adverse experiences and unsafe circumstances can place a great burden on their mental health and psychological well-being [8, 9]. Also amongst the next generation of refugees, i.e. children who were born after their parents fled to another country, research has shown an increased risk of psychological stress [10]. This is described as intergenerational trauma in which traumatic experiences in one generation affect the health and well-being of their children. As such, experiences of children from refugee families (either fled themselves or their parents) are unique in the frequency and kind of adverse experiences they have dealt with. Children also differ in how resilient they are to deal with these adversities [11]. This results in a great variety in to what extent adversities prior, during and after their (parents’) flight journey affect refugee children’s well-being.

There is ample evidence that the adverse experiences and prolonged stress that refugees may have encountered during several stages of their flight journey can result in various emotional, behavioral, cognitive and physical difficulties [8, 9, 12, 13]. Systematic reviews concerning the mental health of child and adolescent refugees and asylum seekers found high prevalence rates for post-traumatic stress disorder (PTSD), depression, anxiety and various internalizing and externalizing problems [8, 9]. In the classroom specifically, this can manifest in different ways. For instance, teachers report explosive anger, inability to concentrate, rule testing, withdrawal, age inappropriate behavior and lower academic achievement [14]. Nevertheless, many refugees exhibit good psychological functioning, despite the numerous stressors during the various phases of the refugee journey [11]. Protective factors, such as a positive self-image and having a support network including social relations with others can have a buffering effect, meaning that children might be less affected by adversity and stress [11, 15]. This buffering effect was also found in empirical studies among refugee children [e.g. 16, 17]. It is therefore argued that a resilience-focused perspective on mental health is essential for mental health support [11, 15], of which trauma-informed teaching practices could be seen as an example [e.g. 18].

The current study is driven by concerns from educational practice. Teachers of young refugee students in the Netherlands reported their worries about the well-being of these students. Many teachers suspect underlying trauma or distress but do not possess the knowledge and skills to recognize trauma [5]. Limited research has been conducted on the well-being of young refugee students in kindergarten and early years of primary school specifically. Moreover, most studies on refugee well-being exclusively rely on psychopathological assessments and neglect the buffering effect of protective factors [11]. In the current study, we aim to serve the need to investigate the school-related well-being of young refugee students aged 4–8 years in the Netherlands, and investigate what particular difficulties (risk behaviors) and strengths (protective factors) are observed among them. We addressed the following research question:

1. To what extent do young refugee students in the Netherlands differ from their non-refugee peers in terms of psychological well-being?

We hypothesize the psychological school-related well-being to be lower among students with a refugee background as compared to students without refugee background. Psychological well-being was assessed in a comprehensive way, including indicators of both psychological and behavioral problems, and several protective factors and strengths. In this way we aim to gain more insights into what particular difficulties and strengths are experienced among refugee children. The research question will be addressed in two ways. First, we examine whether refugee students demonstrate more behaviors associated with several psychological and behavioral problems, and less strengths and protective factors. Second, we studied whether refugee students more often have scores in the clinical range of these psychological and behavioral problems, and in the clinical range of strengths and protective factors, compared to their non-refugee peers.^1^

## Method

### Participants

Data of 542 4- to 8-year-old students were collected, of which 136 students with a refugee-background and 406 non-refugee Dutch students as a control group (see Table 1). The mean age was 6 years and 4 months.

**Table 1:** Demographic characteristics of the samples

		Refugee sample		Non-refugee sample	
		*n*	%	*n*	%
**Gender** ^a^					
	Female	76	55.9	207	51.1
	Male	60	44.1	198	48.9
**Age, years** ^a^					
	4	23	16.9	73	18.0
	5	34	25.0	100	24.7
	6	32	23.5	84	20.7
	7	22	16.2	79	19.5
	8	25	18.4	69	17.0

^a^ Data was missing for one participant from the non-refugee sample

#### Refugee Sample

Using the term refugee student, we refer to students whose teachers and parents identified them as having a refugee background. The sample therefore included students with different legal statuses, and included both students who were born in another country and had fled to the Netherlands (*n* = 116), as well as students who were born shortly after their parents fled to the Netherlands (*n* = 19).^2^ The mean length of residency in the Netherlands from the 116 first generation refugee students was 16 months (range 1–74 months).^3^ They came from 28 different countries, most students being from respectively Syria (*n* = 34), Turkey (*n* = 20) and Iran (*n* = 8). Other countries of birth represented were, among others, Eritrea, Iraq, Pakistan, Venezuela, Lebanon and Afghanistan. The remaining 19 students were born in the Netherlands after their parent(s) fled to the Netherlands before the child’s birth. Different legal statuses were represented in the sample: 65 were in the asylum request or family reunification procedure, 43 were granted a temporary or non-temporary asylum residence permit, three were rejected for asylum, five had a Dutch/European passport most likely because of naturalization (of their parents), and 20 parents did choose not to answer this question.

The students with a refugee background were recruited via 19 primary schools across different regions in the Netherlands. Depending on the municipality, education for refugees, asylum seekers and other newly arrived migrants is organized in different ways. In our sample, 78 students attended education at a school connected to an asylum center (either located on or nearby the premise of an asylum center), 34 students were from schools with multiple newcomer groups for both children from the asylum center and other newcomer children living in the municipality, and 24 students attended regular primary schools, often combined with a special educational program such as an intensive language program in smaller groups several times a week.

#### Non-Refugee Sample

Students of the non-refugee sample were all born in the Netherlands and their parents did not have a refugee background. Most students had parents who were born in the Netherlands (*n* = 337), and 68 students were second generation immigrants with at least one of their parents being born outside the Netherlands. For one participant, information about parents’ country of birth was missing. Data was collected via 67 teachers from 31 primary schools from different regions in the Netherlands.

## Instruments

Three different teacher report questionnaires were used to provide a comprehensive overview of students’ well-being at school: Strengths and Difficulties Questionnaire (SDQ), Social-Emotional Questionnaire (SEV), and Risk and Protective factors Trauma Observation School Situations (RaPTOSS). Although several scales of these instruments are correlated with each other [19] and partly measure similar constructs, in the current study we use these instruments in a complementary way. As such, the international SDQ is used for a general assessment of student well-being which combines several measures of socio-emotional functioning, the SEV scales are used for a more differentiated assessment of well-being, and the RaPTOSS is added for the specific assessment of trauma-related behavior at school.

### Strengths and Difficulties Questionnaire (SDQ)

The Dutch SDQ Teacher Report T4-17 years [20] is a screening instrument for teachers to assess students’ behavior over the last six months on a three-point scale (range 0–2). It consists of 25 items generating five scales: emotional symptoms, peer relationship problems, conduct problems, hyperactivity, and prosocial behavior. We report the total difficulties score and the prosocial behavior scale. The total difficulties score is calculated by summing the scales emotional symptoms, peer relationship problems, conduct problems, and hyperactivity. Scale reliabilities (α) in our sample were acceptable to good for these scales (from .77 to .81).

### Social-Emotional Questionnaire (SEV)

The SEV is a Dutch questionnaire that is widely used in education and youth care in the Netherlands to screen for various social-emotional and behavioral problems among children aged 4 to 18 years [21]. The SEV asks 72 questions to rate the extent to which symptoms have been observed on a five-point scale from never to very often (range 0–4). It assess symptoms of ADHD (18 items), problematic social behavior (26 items), anxious and mood disturbing behavior (18 items), and autistic behavior (10 items). Scale reliabilities (α) in our sample ranged from .81 to .95.

### Risk and Protective Factors Trauma Observation School Situations (RaPTOSS)

The RaPTOSS [19, 22] is a Dutch instrument for teachers assessing the frequency of observable trauma-related behavior of their students in the classroom over the past two weeks. It consists of 29 items assessing risk factors (behavioral indicators of post-traumatic stress), and 25 items assessing protective factors. Risk factors were behavioral indicators of PTSD as described in the DSM: intrusion symptoms, avoidance of trauma-related stimuli, negative alterations in cognitions and mood, alterations in arousal and reactivity, and dissociation. Protective factors were safety and relations, self-regulation, self-image, and everyday life. Answer options all range from not applicable to very often applicable (range 0–3). We report scale scores for the five risk factors and four protective factors separately, and sum scores for the risk factors (range 0–87) and protective factors (range 0–75), where high scores on risk factors should be interpreted negatively (many psycho trauma symptoms), and high scores on protective factors should be interpreted positively (many protective factors present). Scale reliabilities (α) in our sample ranged from .71 to .95.

### Design and Procedures

This study was part of a larger study on the well-being and second language learning of young students with a refugee background. Teachers were recruited through email, social media and the personal networks of the researchers. Parental permission of students was obtained via teachers with information letters and informed consents, for the refugee students available in different languages (Dutch, English, Arabic, Persian, Tigrinya, Turkish, Spanish) and complemented with icons. For the refugee students, background information (as described in participants section) was collected via a parental questionnaire in the available languages. Teachers filled in the SDQ, SEV and RaPTOSS, which took around 30 minutes to complete for one student. These questionnaires were completed in Excel (72%) or on paper (28%). Data collection took place between 2019 and 2021. The study was approved by the Ethical Committee of Psychology of the University of Groningen (PSY-1819-S-0012 and PSY-1920-S-0014).

### Data Analysis

A MANOVA was performed to assess whether the well-being of students with a refugee background was different from the non-refugee control group. Pillai’s Trace test statistic was used as the most robust MANOVA test statistic to account for the unequal sample sizes of the group and violation of the homogeneity of variance assumption for several dependent variables [23]. The univariate ANOVAs as part of the model demonstrated on which specific well-being measures the groups differ. We report partial eta squared (partial η2) with 90% confidence intervals as effect size measure. Partial eta squared values were interpreted according to Cohen’s benchmarks [24], where η2 = 0.01 indicates a small effect, η2 = 0.06 represents a medium effect, and η2 = 0.14 is considered a large effect.

Categorical Chi-square analyses were conducted to examine differences between students with and without refugee background in relation to the categorization of scores in the normal or (sub)clinical range of the psychological well-being measures. Raw sum scores on the SDQ, SEV and RaPTOSS were categorized according to the cut-off scores described in the manuals, which were based on Dutch (SEV and RaPTOSS) and British (SDQ) reference groups. For the sake of brevity and comparability of the outcomes concerning the SDQ, SEV and RaPTOSS, we used a twofold classification, with an aggregated category of scores in the ‘clinical range’ referring to all scores ≥ 80th percentile, and scores in the ‘normal range’ referring to all scores < 80th percentile. For the positively formulated scales (SDQ prosocial behavior and RaPTOSS trauma protective factors), the clinical range encompasses all scores ≤ 20th percentile, indicating that students demonstrated minimal prosocial behavior or had limited protective factors of trauma. For the RaPTOSS, we adhered to the norms for the sum scores of all trauma risk factors, and trauma protective factors instead of the specific subscales. We report odds ratios (OR) with 95% confidence intervals as effect size measure.

Analyses were performed in SPSS version 28, and 90% confidence intervals for partial eta squared were calculated in R using the MBESS package. To correct for family-wise error rates, adjusted *p*-values were calculated [25] using the sequentially rejective Holm-Bonferroni procedure [26].

## Results

**Fig. 1:**
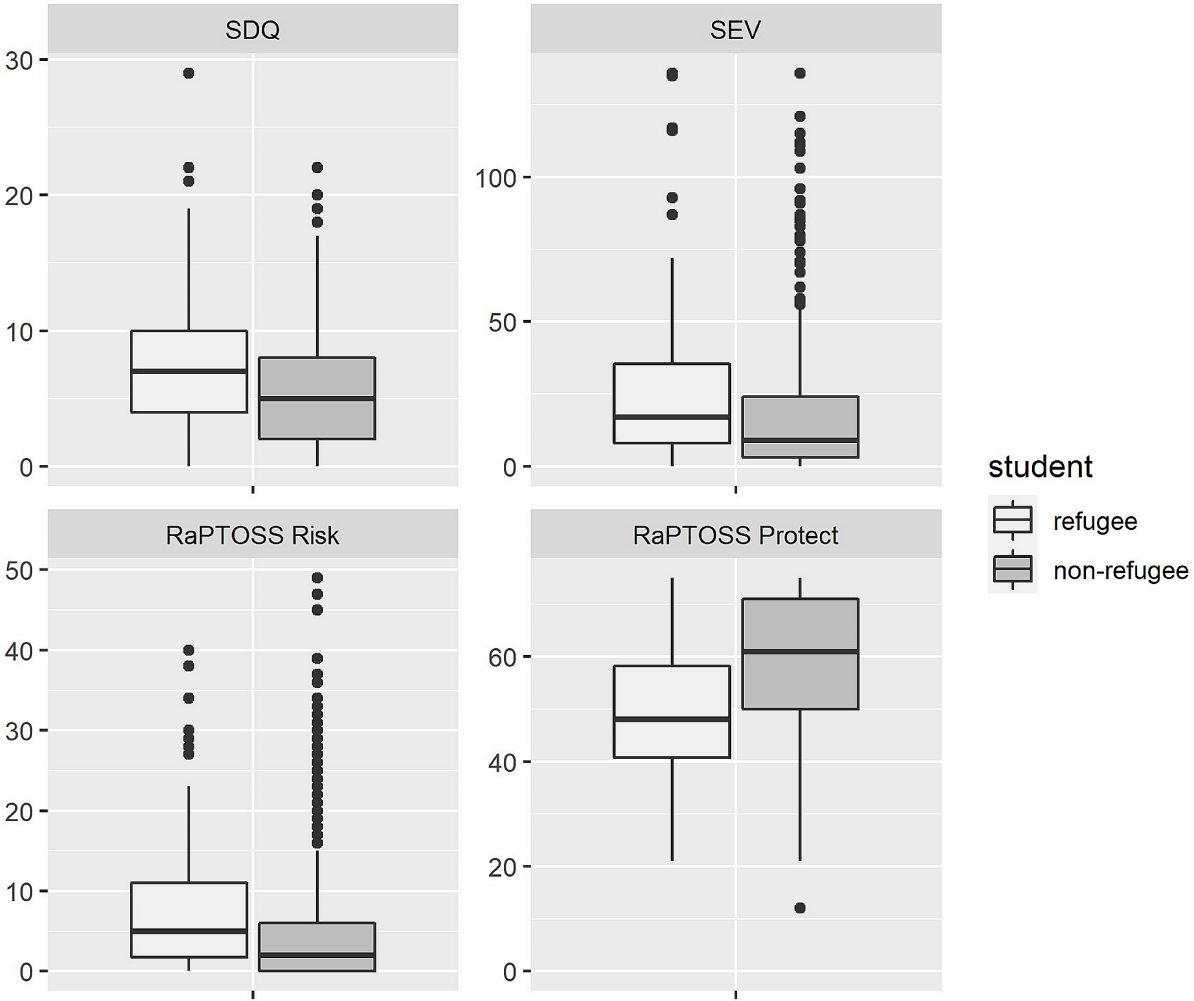
Well-being scores (SDQ, SEV, RaPTOSS risk and RaPTOSS protect) for students from the refugee and non-refugee samples. *Note*. To provide a general impression of well-being differences, we used the total difficulties score for the SDQ, and a total score for the SEV (adding all scale scores)

Figure 1 provides a general impression of the well-being of students with and without a refugee background through comparing sum scores of the SDQ, SEV and RaPTOSS. Results demonstrated a significant difference in overall psychological well-being, where students with a refugee background had a lower well-being compared to the non-refugee control group, *F* (16, 523) = 8.887, *p* < .001, Pillai’s Trace = 0.214, partial η2 = 0.214. Zooming in to the scales as assessed with the SDQ, SEV and RaPTOSS, results of the univariate ANOVAs are shown in Table 2. Significant differences were found between the refugee and non-refugee samples on most, 13 out of 15, well-being measures, except autistic behavior (SEV) and avoidance of trauma-related stimuli (RaPTOSS). The effect sizes indicated mostly small effects, and medium effects for the protective factors self-image, self-regulation, everyday life and prosocial behavior. In sum, more psychological and behavioral problems, and less prosocial behavior and trauma protective factors were observed among the students from the refugee sample compared to the non-refugee sample.

**Table 2:** Mean and standard deviation (SD) across groups and univariate ANOVAs with effect sizes (partial η2)

Measures		Refugees (*n* = 135) ^b^		Non-refugees (*n* = 405) ^c^		*F* (1, 538)	*p* ^a^	partial η2	90% CI for partial η2	
		*M*	*SD*	*M*	*SD*				*LL*	*UL*
**SDQ**										
	Total difficulties	7.65	5.19	5.83	5.16	12.575	< .003**	.023	.007	.048
	Prosocial behavior	7.05	2.12	8.25	1.93	34.272	<.001***	.060	.031	.095
**SEV**										
	ADHD symptoms	11.86	13.08	8.15	11.64	9.680	.012*	.018	.004	.040
	Problematic social behavior	6.24	9.54	4.09	7.55	7.152	.025*	.013	.002	.033
	Anxious and mood disturbing behavior	5.54	5.80	3.62	4.89	14.137	.002**	.026	.008	.051
	Autistic behavior	2.66	3.61	1.94	3.62	3.971	.094	.007	.000	.024
**RaPTOSS trauma risk factors**										
	Intrusion	1.60	1.99	1.11	1.75	7.502	.025*	.014	.002	.034
	Avoidance of trauma-related stimuli	0.89	1.92	0.75	1.84	0.542	.462	.001	.000	.010
	Negative alterations in cognitions and mood	1.63	2.31	0.98	1.75	11.952	.004**	.022	.006	.046
	Alterations in arousal and reactivity	2.16	2.80	1.31	2.20	12.911	.003**	.023	.007	.048
	Dissociation	1.88	2.53	1.17	2.34	9.004	.014*	.016	.003	.039
**RaPTOSS trauma protective factors**										
	Safety and relations	15.83	4.00	17.59	3.87	20.571	<.001***	.037	.015	.066
	Self-regulation	9.41	3.81	12.60	4.56	53.876	<.001***	.091	.056	.131
	Self-image	9.78	4.39	13.13	4.15	63.915	<.001***	.106	.068	.148
	Everyday life	14.13	3.08	16.13	2.71	50.959	<.001***	.087	.052	.126

a Holm-Bonferroni adjusted *p* values

b One participant of the refugee sample was not included due to missing data on the SDQ

c One participant of the non-refugee sample was not included due to missing data on the SEV

*p < .05. **p < .01. ***p < .001

The relative frequencies of refugees’ and non-refugees’ scores in the clinical range on the different well-being measures are displayed in Table 3. Chi-square tests, also displayed in Table 3, demonstrated that students with a refugee background compared to those without a refugee background were significantly more likely to have scores in the clinical range for prosocial behavior (SDQ), problematic social behavior (SEV), and risk and protective factors of trauma (RaPTOSS). The odds ratios demonstrated that these differences between the students with and without a refugee background were largest for trauma protective factors. Students with a refugee background were not more likely to have scores that fall into the clinical range of the SDQ total difficulties score, ADHD symptoms (SEV), anxious and mood disturbing behavior (SEV), and autistic behavior (SEV) compared to students without a refugee background. Overall, 49.3% (*n* = 67) of the refugee sample had at least one score in the clinical range for all psychological and behavioral problems assessed (total difficulties, ADHD, problematic social behavior, anxious and mood disturbing behavior, autistic behavior, and trauma risk factors), compared to 27.8% (*n* = 113) of the non-refugee sample. This means that a little more than half of the refugee sample (50.7%, *n* = 69) did not have any scores in the clinical range for all psychological and behavioral problems assessed (total difficulties, ADHD, problematic social behavior, anxious and mood disturbing behavior, autistic behavior, and trauma risk factors), compared to 72.2% (*n* = 293) of the non-refugee sample. In addition, 60.3% (*n* = 82) of the refugee students did have at least one score in the clinical range for prosocial behavior and trauma protective factors, compared to 23.6% (*n* = 96) of the non-refugee students.

**Table 3:** Relative frequencies in clinical score range and 2 × 2 chi-square test on well-being measures in refugee and non-refugee samples

Measures		% in clinical score range		*χ*² (1)	*p* ^a^	OR	95% CI	
		Refugee sample (*n* = 136)	Non-refugee sample (*n* = 406)				*LL*	*UL*
SDQ ^b^								
	Total difficulties	19.1%	15.3%	1.085	.454	1.31	0.79	2.17
	Prosocial behavior	26.5%	11.9%	16.589	<.001***	2.68	1.65	4.35
SEV ^c^								
	ADHD symptoms	14.8%	8.6%	4.256	.117	1.84	1.02	3.32
	Problematic social behavior	16.3%	7.4%	9.252	.012*	2.44	1.34	4.40
	Anxious and mood disturbing behavior	14.8%	7.6%	6.116	.054	2.01	1.15	3.83
	Autistic behavior	15.6%	11.6%	1.460	.454	1.41	0.81	2.45
RaPTOSS								
	Trauma risk factors	38.2%	20.9%	16.142	< .001***	2.34	1.54	3.56
	Trauma protective factors	51.5%	19.0%	54.457	< .001***	4.53	2.98	6.88

Note. OR = odds ratio; CI = confidence interval; LL = lower limit; UL = upper limit

*p < .05. **p < .01. ***p < .001

^a^ Holm-Bonferroni adjusted p values

^b^ Data was missing for one participant from the non-refugee sample

^c^ Data was missing for one participant from the refugee sample

## Discussion

The aim of the current study was to examine the psychological well-being of 4- to 8-year-old students with a refugee background in the Netherlands, compared to Dutch students without a refugee background. The results of the current study support the hypothesis that young students with a refugee background in the Netherlands had an overall lower psychological well-being than their peers without a refugee background. We first assessed whether teachers observed more behaviors associated with a number of psychological and behavioral problems, and less strengths and protective factors among the students with a refugee background compared to students without a refugee background. The results showed that more total difficulties in socio-emotional functioning, and more behaviors associated with post-traumatic stress disorder, anxious and mood disturbing behavior, problematic social behavior and ADHD symptoms were observed among students with a refugee background compared to non-refugee students. With regard to the protective factors and strengths, students from the refugee sample had significantly lower scores on prosocial behavior, safety and relations, self-regulation skills, self-image and a stable everyday life. Additionally, it was also investigated whether refugee students more often had scores in the clinical range of psychological problems compared to their non-refugee peers. The findings demonstrated that this was the case for problematic social behavior and post-traumatic stress behaviors. In the domain of prosocial behavior and protective factors of trauma, students with a refugee background were significantly more likely to have scores in the clinical range compared to their non-refugee peers. While all these results imply that, overall, students with a refugee background are at increased risk of low well-being, the findings also demonstrate that around half of the students from the refugee sample did not have any scores that usually fall in the clinical range of the psychological and behavioral problems assessed. Yet, the largest differences we found between students with and without a refugee background were in the domain of strengths and protective factors.

These results are in line with the worries from educational practice where teachers raised concerns about the well-being of young refugee students, which was an important motive for investigating this topic [5]. The pattern of results is also consistent with previous literature on refugee well-being [6–9, 12, 13]. Some refugee children develop well, but overall, students with a refugee background are at increased risk of reduced well-being, which is also observed by teachers in the classroom. The findings of our study extend these findings to a younger refugee sample aged 4–8 years, as prior research mostly focused on older children, and none of them particularly focused on students in kindergarten and early years of primary school. Moreover, so far, most studies on refugee well-being have exclusively focused on prevalence of psychopathology, and much less their behavior in the classroom [11]. What sets this study apart is its examination of psychological and behavioral problems, but also protective factors and strengths, within the unique context of the classroom. The findings of the current study showing that the largest differences between students with and without a refugee background were in the domain of protective factors, is an important contribution to existing literature.

## Implications

The findings have some potential intervention implications. Given the link between protective factors and psychological well-being [16, 17], our findings highlight the need for support (programs) to promote protective factors among all students with a refugee background. Teachers can have direct influence on some of the protective factors and can address these in their pedagogy. For instance, teachers can facilitate positive teacher-student and student-to-student interactions in the classroom. They can also encourage a positive self-image of students, and help students to regulate stress and improve self-regulation skills. These strategies align well with the principles of trauma-informed teaching [27, 28]. Another important factor in trauma-informed teaching is for teachers to be aware of the possible impact of stress and potential trauma on student’s behavior. Instead of interpreting problematic behavior as rule-breaking, it should be seen as a sign that the students may feel unsafe. Such feelings of unsafety or struggling with stress or traumatic events may also distract students from learning tasks [29, 30]. As such, teachers understanding the potential impact of trauma and stress on both student behavior and learning processes is an important starting point to better support refugee students.

## Limitations and Future Research

This study has several limitations. First, our data relied on teacher reports in the specific context of school-related well-being. The student’s own experiences and perceptions and their parents’ perspective were not taken into account in the current study. In practice, teachers often serve as a key informant to signal problematic behavior among their students. However, it should be noted that teachers are not trained as psychologists and therefore teacher reports only should not be used to diagnose students. Second, the distinction of students’ scores belonging to the normal versus clinical range was solely based on the statistical method of percentile scores retrieved from other populations. We did not conduct diagnostic interviews, and we did not take into account the severity of functional impairment from the student’s perspective. Naturally, the clinical scores on these measures alone do not yield information to diagnose students with mental disorders and should not be used as such. However, from a school psychologist perspective, scores in the clinical range have a signaling function for monitoring the student’s well-being and provide potential support. A third limitation concerns the cross-cultural validity of the questionnaires and corresponding norm scores. Norm scores were based on Dutch (SEV and RaPTOSS) and British (SDQ) reference groups, and since previous studies have shown cultural differences in the manifestation of psychopathology [31], it could be questioned whether the instruments are suitable for the diverse group of refugee students in this study. A final limitation concerns the heterogeneity of the refugee sample with students from many different countries and with different legal statuses in the host country. It may be that well-being differs between subgroups of students in the refugee sample, but the sample was too small to conduct such analyses. It is also conceivable that student well-being is related to the type of education students attend. For instance, it could be that teachers from schools with many students with a refugee background are better trained to deal with trauma-related behavior in the classroom. Nevertheless, this study provides a more representative view on the well-being of the diverse group of students with a refugee background in kindergarten and early years of primary education. In terms of future research, the current findings should be extended by examining potential differences between various refugee populations and types of (newcomer) education, and assess the complex interplay between mental health problems and protective factors on different ecological levels in longitudinal research.

## Summary

This study examined the psychological well-being of 4- to 8-year-old students with a refugee background residing in the Netherlands, compared to Dutch peers without a refugee background. Both psychological and behavioral problems, and strengths and protective factors were assessed using questionnaires that were completed by students’ primary school teachers. Results showed that anxious and mood disturbing behavior, ADHD symptoms, problematic social behavior and post-traumatic stress symptoms were more often observed among students with a refugee background compared to their non-refugee peers, which is in line with previous research on psychological distress among refugee youth. The largest differences though between students with and without a refugee background were that refugee students had less self-regulation skills, a more negative self-image, a less stable everyday life, less positive relations, and demonstrated less prosocial behavior in the classroom. Teachers can contribute to enhancing refugee students’ well-being through promoting these protective factors.

## Data Availability

The anonymized data and statistical code that support the findings of this study is openly available on https://osf.io/8byst/

## Footnotes

1 Using the term ‘clinical range’, we refer to scores that either exceed or fall below a cut-off score derived from percentile scores or other populations. Scores in the clinical range for psychological and behavioral problems indicate a higher prevalence of behaviors associated with these problems. Conversely, scores in the clinical range for strengths and protective factors indicate a limited presence of these behaviors.

2 Country of birth is unknown for one student.

3 Length of residency in the Netherlands is unknown for four students.
